# Recording Large Extracellular Spikes in Microchannels along Many Axonal Sites from Individual Neurons

**DOI:** 10.1371/journal.pone.0118514

**Published:** 2015-03-03

**Authors:** Marta K. Lewandowska, Douglas J. Bakkum, Santiago B. Rompani, Andreas Hierlemann

**Affiliations:** 1 Bio Engineering Laboratories, Department of Biosystems Science and Engineering, ETH Zurich, Basel, Switzerland; 2 Neural Circuits Laboratory, Friedrich Miescher Institute, Basel, Switzerland; University of Genova, ITALY

## Abstract

The numerous connections between neuronal cell bodies, made by their dendrites and axons, are vital for information processing in the brain. While dendrites and synapses have been extensively studied, axons have remained elusive to a large extent. We present a novel platform to study axonal physiology and information processing based on combining an 11,011-electrode high-density complementary metal-oxide semiconductor microelectrode array with a poly(dimethylsiloxane) channel device, which isolates axons from somas and, importantly, significantly amplifies recorded axonal signals. The combination of the microelectrode array with recording and stimulation capability with the microfluidic isolation channels permitted us to study axonal signal behavior at great detail. The device, featuring two culture chambers with over 30 channels spanning in between, enabled long-term recording of single spikes from isolated axons with signal amplitudes of 100 μV up to 2 mV. Propagating signals along axons could be recorded with 10 to 50 electrodes per channel. We (i) describe the performance and capabilities of our device for axonal electrophysiology, and (ii) present novel data on axonal signals facilitated by the device. Spontaneous action potentials with characteristic shapes propagated from somas along axons between the two compartments, and these unique shapes could be used to identify individual axons within channels that contained many axonal branches. Stimulation through the electrode array facilitated the identification of somas and their respective axons, enabling interfacing with different compartments of a single cell. Complex spike shapes observed in channels were traced back to single cells, and we show that more complicated spike shapes originate from a linear superposition of multiple axonal signals rather than signal distortion by the channels.

## Introduction

The classical picture of a neuron is an electrically excitable cell with a large number of dendrites that integrate incoming (analog) signals and compute whether to produce an all-or-none (digital) action potential (AP) that reliably propagates along the axon. Dendritic integration has been extensively researched [1–4], while the axon has often been portrayed as a faithful transmission line. The earliest experiments on axons, performed ex vivo using extracellular electrodes on the spinal cords of invertebrates [5], as well as cats [6], however, provided evidence of more complicated and subtle behavior including differences in propagation velocity and excitability in axonal branches, conduction failure [7,8], and reflection at branch points [9,10]. Recently, there has been renewed interest in axonal properties both along the lines of fundamental research into their information processing capabilities [11–15], as well as in the context of potential medical applications for axonal regeneration following injury [16,17], which are made possible by the development of microfluidic and microelectronic tools [18–20].

The voltage clamp technique, the predecessor to the patch clamp, enabled the first measurements of invertebrate axons and led to the creation of the model of action potential generation and propagation that is still generally accepted [5]. Since then, the patch clamp has been widely adopted and used to discover a rich repertoire of behavior including cell-to-cell communication [21], cell types, dendritic integration [2,22], and much more. Due to the very small size of mammalian cells, the objects that could be patched were often limited to somas and dendritic spines, so that prevailingly somas and dendrites were studied using the patch clamp. Mammalian axons, that can be as small as 0.2 μm in diameter, are virtually impossible to patch while intact, and are, therefore, difficult to study. Additionally, there is a natural limitation on the proximity and number of patch clamp electrodes that can be used at any one time, limiting the number of cells or the number of points on a single cell that can be simultaneously measured. The adaptation of micro-fabricated devices to biology has facilitated the study of ever-smaller isolated living systems in a controlled environment. These new tools bypass some of the limitations of the patch clamp by enabling long term recordings, over weeks and even months, of many individual cells, and even cell processes, at the same time.

A number of groups have, therefore, attempted to isolate and study axons in vitro by using substrate patterning ranging from chemo-attractants to conducting polymers [23–27], as well as physical barriers that confine somas to compartments and direct axonal growth [28]. For example, the group of Jeon developed and commercialized (Xona Microfluidics LLC) the archetypical two-chamber, multi-channel device that has been adopted by a number of other groups [19]. The basic design comprises two fluidically-isolated culture chambers and media wells connected by microchannels into which only neurites can grow: the channel dimensions are designed to be too narrow and low for neuronal cell bodies to enter [29]. Other systems include that by Folch’s group, who developed a microfluidic micro jet system that combined channels for neurites growing from ex-planted tissue with a controlled chemical gradient system [30],[31]. Peyrin et al. created ‘axon diodes,’ channels that narrow at one end, and orient the network in one direction [32]. Wieringa et al. explored, how channel size influences axonal outgrowth and how bundles may be separated by cleverly designed bifurcations [33]. These systems were used in combination with microscopy.

The combination of extracellular electrodes with microchannels allowed electrophysiology to be performed on somas located outside of channels and on axons located inside of channels. Importantly, isolating the axons in channels provided an important advantage over open cultures: a significant amplification of the axonal signal caused by the increased extracellular sealing resistance [34,35]. Claverol-Tinturé built relatively cheap and easy-to-use devices that could house single somas and read out signals from either somas or axons [36,37]. Dworak and Wheeler created a five-compartment structure interconnected by channels placed on top of several long electrodes that monitored action potential propagation direction and velocity [38]. A number of groups have integrated commercial multi-electrode array (MEA) systems featuring 60 low-density electrodes with commercial or custom microfluidic devices in order to functionally connect cultures of either one cell type or to create co-cultures of different cell types [39–41]. Brewer et al. demonstrated self orientation when co-culturing different areas of the hippocampus [41].

All of these previous approaches were limited in resolution by the number of channels or the number and size of available electrodes. Recorded signals were either from large cell populations, making it impossible to distinguish between individual cells, or from a single cell, but utilizing just a single electrode. In the present work, we increase resolution by merging two technologies to study axon electrophysiology in an unprecedented way: custom poly(dimethylsiloxane) (PDMS) devices containing more than 30 microchannels, combined with custom complementary metal-oxide-semiconductor (CMOS)-based high-density MEAs (HDMEAs) containing 11,011 densely-arranged platinum electrodes. The goals of this paper are two-fold. First, we explain our device assembly and evaluate its performance and added capabilities for extracellular axonal physiology. Second, we take advantage of the increased detail of data facilitated by the device to clarify an issue in the field: complex action potential waveforms often seen in microchannels are not an artefact from the channels themselves, but instead arise from a linear superposition of signals from different axonal branches. Importantly, this result helps to validate experiments that use microchannels for axonal electrophysiology. Moreover, our device’s sub-cellular spatial resolution, as a result of the high electrode density, and its large number of channels enabled a high measurement throughput, which led to a better understanding of how channels affect extracellular axonal signals. As expected, the channels amplified AP signals 20-fold and up to more than 150-fold, which then saturated the recording amplifiers. Most amplified axonal signals had a stereotypical positive-first, tri-phasic shape, but more complex waveforms were also observed, whose origin can now be explained. Spontaneous and stimulus-evoked spikes were used to correlate somas with their respective axonal segments, axonal spike evolution was observed over many days, and complex spikes could be associated to single cells. The presented device and methods have given us access to axonal signaling in a way that was not possible before.

## Materials and Methods

### Microelectrode array chip

Details of the design, fabrication, and characterization of the high-density microelectrode array (HDMEA) can be found elsewhere [20]. Important features of the HDMEA include: an array of 11,011 platinum electrodes at a density of 3161 electrodes per mm^2^ (electrode size: 6 x 8 μm^2^, center-to-center pitch: 17 μm) housed on the same chip with three-stage amplification, signal conditioning, analog-to-digital conversion, and stimulation buffers. A subset of 126 electrodes can be read out simultaneously and can be reconfigured and rerouted within 1.4 ms [20,42,43]. Each electrode of the HDMEA can be routed to one of the two stimulation buffers.

### Soft lithography and chip packaging

Moulds for the PDMS pieces were made using standard soft lithography with SU-8 on silicon wafers. To create the channels, SU-8 3005 (MicroChem, USA) was spin coated onto a four-inch silicon wafer at 3500 rpm for 30 s, or 6000 rpm for 5 min, depending on the desired height. The SU-8 was soft baked at 95°C for five min, exposed to UV on a Mask aligner (Süss MicroTec AG, Germany) for 5 s, and then baked again. Development was performed using mr-Dev 600 (micro resist technology GmbH, Germany) for five min. Resulting structures were seven (3500 rpm) or four (6000 rpm) μm in height. Following a hard bake, the second layers were spun on. Culture chambers were made using two layers of SU-8 100: the first layer was spun on (1000 rpm for 30 s) and then baked, and then the second layer was directly spun on after cooling of the first layer. Following the second cooling, the SU-8 was exposed for 30 s, and then baked again. Development was performed upside down for 30 min. The resulting culture chambers were 700 μm in height.

PDMS culture chambers were open at both ends to facilitate medium and oxygen exchange. PDMS (Sylgard 184, Dow Corning) was poured onto the structured wafers and allowed to redistribute for 1 h. It was cured at 60–80°C for at least 4 h.

**Fig 1:**
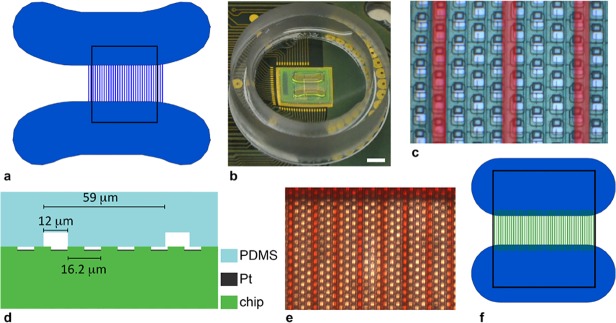
Geometry and layout of poly(dimethylsiloxane) axonal channel device on microelectrode array chip (a) Drawing of axonal channel device, with 12 μm channels, and neuronal culture chambers; rectangle shows dimensions of electrode array, which is 1.75 x 2.0 mm^2^. (b) Photograph of packaged chip wire-bonded to a printed circuit board and with channel device on top, scale bar is 2 mm, the plastic ring is 18 mm in diameter and used to hold cell medium. (c) Zoom in on electrodes and channels: channels are lighter and highlighted in red; scale bar is 10 μm. (d) Cross section of chip, electrodes and channel device on top showing characteristic dimensions and corresponding to c. (e) Photograph of channel region of device; channels were filled with red food coloring to illustrate tightness of bonding to chip surface; scale bar is 20 μm. (f) Smaller chambers and channels used for some experiments. Channels were 2–8 μm in width; black box corresponds to array, which is 1.7 x 2.0 mm^2^.

The HDMEA chip was glued to a custom PCB and bonded using an automatic wire bonder (Esec AG, Switzerland). A ring made of poly(methyl-methacrylate) (PMMA) to hold cell medium was glued to the PCB. The PDMS cast was cut into pieces to yield the individual chamber and channel devices, which were cleaned with isopropanol and blown dry with N_2_ gas. The chip surface was also cleaned with isopropanol and blown dry. The PDMS device was placed onto the array with a pair of tweezers and pushed down. As Fig. 1C shows, a first design was such that careful alignment between the HDMEA and PDMS device was not necessary since channel size was chosen to be larger than electrode size, and the channel pitch was not a multiple of the electrode pitch.

To protect the bond wires, PDMS was poured into the space between the PDMS device and the PMMA ring and allowed to cure. The entire packaged chip is shown in Fig. 1B: a close-up view of the entire electrode array and the electrodes covered with the PDMS channel device. Fig. 1F shows a later version of the PDMS device, which included smaller culture chambers and narrower channels (down to 2μm width).

### Readout protocols

Many different electrode arrangements were used for recording. Up to 126 electrodes could be read out at any given time, and switching between arrangements happened in 1.4 ms, so new configurations were loaded many times during a recording session. Typical strategies for monitoring both spontaneous and evoked activity included (i) using pre-set block configurations to cover either the entire chip or specific areas of interest, i.e., a cell body and its processes, or (ii) generating many random configurations to sample the chip space. In both cases, recordings in each configuration tended to last 60–120 s.

### Stimulation protocols

Soma and axons were stimulated with biphasic positive-first voltage pulses of 200 ms in width using the on-chip stimulation buffer. Proper stimulation voltages were established by starting stimulation at a low voltage (100 mV) and manually stepping through voltage differences of 50–100 mV, until a response was evoked. Successful stimulation was achieved upon applying between 250 mV up to 1.1 V, depending on electrode location.

Stimulation movies, such as the S1 Movie, were made using the following protocol. A configuration of 100 random readout electrodes and one fixed stimulation electrode was routed, and 30–50 voltage pulses were applied at a frequency of ∼3 Hz. After each pulse, the raw data was recorded for a preset amount of time, generally 10–15 ms. This was repeated for 90–100 different configurations in order to record the response on the whole array to stimulation of the fixed electrode. The data was put together in software by plotting the median-average for every electrode. Stimulations were performed both with and without synaptic blockers. Reliable directly-evoked responses were the same regardless of the presence of blockers [44].

### Chip functionalization

Prior to cell plating, the chip was activated using plasma oxygen, which sterilizes the chip before the introduction of cells, and causes the PDMS to become hydrophilic, facilitating filling of the channels. Poly(ethyleneimine) (0.05% wt., in borate buffer, Sigma-Aldrich), used as an attachment promoter, was incubated on the chip for 45 min and then rinsed off before adding 0.02 mg mL^-1^ laminin (Sigma-Aldrich) and incubating at 37°C for 30 min. Laminin was vacuumed off just prior to cell plating, leaving behind a thin layer.

### Neuronal cell plating

Permission to perform animal experiments was granted under animal license 2358, approved by the Basel-City Cantonal Veterinary Authority. Primary neurons were isolated from embryonic day 18 (E18) Wistar rats in accordance with Swiss federal laws on animal welfare. Timed pregnant females obtained from Charles River Laboratories, France were anaesthetized with isofluorane. The female was immediately sacrificed with a guillotine and embryos were extracted. Embryos were sacrificed by severing the spinal cord. Their primary cortices were removed and placed into Hank’s Balanced Salt Solution free from Mg^2+^ and Ca^2+^.

Cortices were dissociated chemically in 0.25% trypsin with ethylene-diamine-tetra-acetate (EDTA) for 15 min at 37°C. The trypsin was washed away, and then tissue was mechanically dissociated by trituration using a pipette tip. Cells were filtered to exclude clumps of tissue, and, then, the single cells were counted. Typical plating densities were 10k to 20k cells per culture chamber or 1,300–2,600 cells per mm^2^. Cells were kept in Neurobasal-based plating medium (Neurobasal supplemented with horse serum from HyClone, GlutaMAX, and B27) for 24 h, and, then, medium was replaced with Dulbecco’s Modified Eagle Medium (DMEM)-based medium (DMEM supplemented with horse serum, GlutaMAX, and sodium pyruvate). Medium was changed once a week. Experiments were conducted in a humidified incubator (RH 65%) at 36°C and 5% CO_2_. The PMMA ring was sealed with a second ring to protect the cultures from fungus and bacteria [45]. Chemicals were purchased from Invitrogen unless otherwise noted.

### Confocal microscopy

Fixed and immunostained cells were imaged with a Nikon A1 microscope using NIS elements software. Images were taken in z stacks and stitched together to create high-resolution mosaics. Image processing was done with Fiji software including the plug-in MosaicJ [46]. For immunostaining protocol, please see S1 Text.

## Results

### PDMS channel device was complementary to chip

The platform is shown in Fig. 1. The schematics in Fig. 1A and 1F demonstrate the size and layout of the culture chambers and axon channels in relation to the electrode array. The culture chambers were designed to be larger than the array to ease cell plating. Channel length was chosen to be greater than 450 μm, since Taylor et al. found this was the minimum length necessary for only axons to grow all the way through channels [47]. Most experiments were performed using the larger culture chambers (5.2 x 1.6 mm^2^) and wider channels (12 μm) shown in Fig. 1A-1, and later experiments were performed with smaller culture chambers (2.5 x 1.0 mm^2^) and narrower channels (2–8 μm) shown in Fig. 1F. After careful cleaning of the CMOS and PDMS surfaces, PDMS sealed well to the CMOS array, as evidenced by Fig. 1E, where red food coloring was introduced into the culture chambers and flowed into the channels. Due to the recessed geometry of the Pt electrodes (Fig. 1D) more than one column of electrodes could be within the same channel when devices with larger channel widths were employed.

### Putative axons grew into channels and high tissue density was observed in entire channels

**Fig 2:**
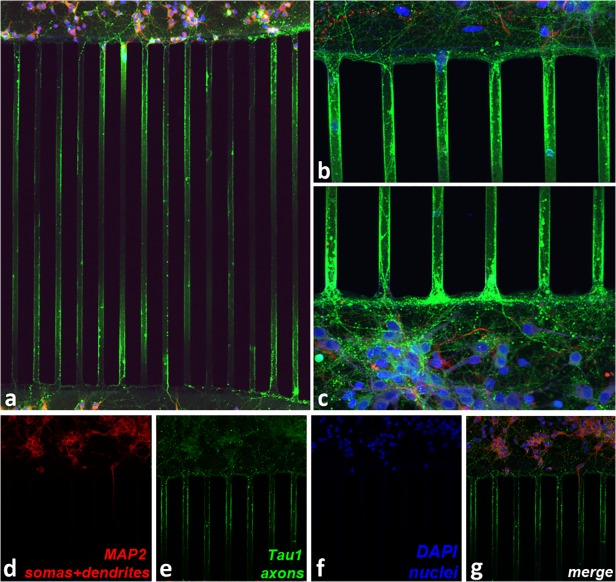
Immunostained cells primarily extended axons into the channels. MAP2 (soma and dendrites) is shown in purple, Tau1 (axons) in green, DAPI (nuclei) in blue, GFAP (glia) in red: (a) Larger image of culture chambers and channels in between, (b) and (c) Close-up views of edges of culture chambers and several channel entrances. Channels were 12 μm wide, and spaced with a pitch of 59 μm. Individual colors showing different cell components: (d) somas and dendrites, (e) axons, and (f) nuclei. (g) Merged image of d-f.

The width of a channel, 2–12 μm, allowed more than one neurite to thread into it. Cells were plated in both chambers, thus neurites grew in both directions. Fig. 2 depicts confocal microscopy images of fixed and stained cells and processes after 7 DIV in one of the PDMS devices bonded to glass. Microtubule associated protein 2 (MAP2), expressed primarily in soma and dendrites, is shown in red; Tau1, expressed primarily in axons, is shown in green; 4',6-diamidino-2 phenylindole (DAPI), a fluorescent dsDNA stain, is in blue; and glial fibrillary acidic protein (GFAP), expressed in astrocytes, is shown in purple. See supporting information (S1 Text) for details about immunostaining. Most cell bodies stayed 20 μm or more away from the channel entrances, and neuronal cell bodies did not enter the channels. Quite clearly, dendrites and axons threaded into the microchannels, but only axons grew all the way through the up to 950 μm long structures. Axons tended to grow straight, guided by features, such as edges. [32] Five successful stainings on chips and six on glass, using the antibody to Tau1, all showed that axons consistently grew into channels.

**Fig 3:**
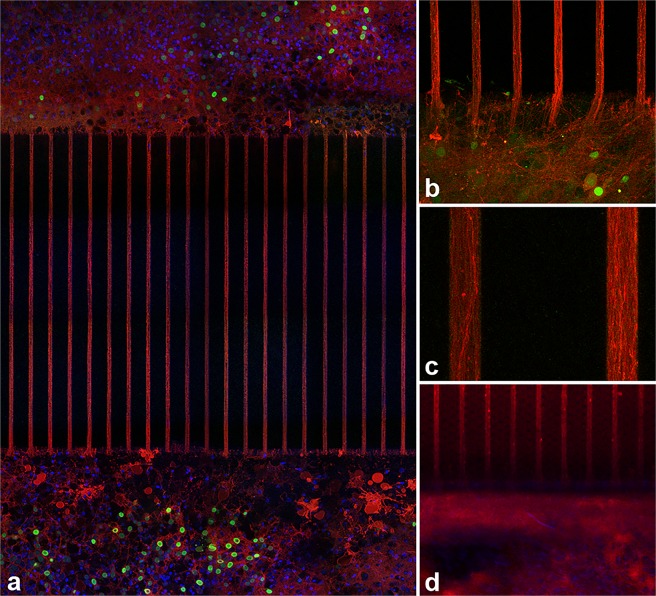
Putative axons, infected with adeno-associated viruses (AAVs), grew through channels. Some neurons were infected with an AAV carrying green fluorescent protein and are shown in green and the cell membrane was infected with an AAV carrying tdTomato and are shown in red. Additional DAPI (dsDNA stain) immunostaining in a and c is in blue. (a) Fixed overview of channels and chambers, (b) Zoomed in, live cell image showing processes growing into channel, (c) Further zoom into channels near middle, (d) Fixed cells on chip imaged through 0.7 mm of poly(dimethylsiloxane).

The visualization of axons in the middle of the channels was difficult, most probably due to the fact that the antibodies did not diffuse all the way into the channels. Therefore, infection with adeno-associated viruses (AAVs) carrying fluorescent proteins was additionally used to live image the large number of axons present throughout the length of the channels and to show that neurite density was consistent along a channel. Fig. 3B shows neurites threaded into the channels, and Fig. 3A shows that the processes grew into and through the channels. The continuity of the fluorescence along the length of a channel is consistent with neurites that crossed fully from one chamber to the other. The close-up view in Fig. 3C, which was taken near the middle of a channel, shows that the channels contained many neural processes, and in spite of the length of the channels, the putative axons were healthy and viable. Fig. 3A-3C all show cultures in PDMS devices that had been bonded to glass for better visualization. Fig. 3D shows a healthy culture on a chip, and was imaged through almost 1 mm of PDMS. An adeno-associated virus (pAAV) that expresses Cre recombinase (Cre) fused to green fluorescent protein (GFP) under the control of the cytomegalovirus (CMV) promoter infected neurons and is shown in green. A second pAAV was induced by Cre via the Double Inverted Open reading frame system (DIO) to express the membrane-targeted red fluorescent protein, tdTomato. The tdTomato was localized to the cell membrane, including all processes (dendrites and axons), and is shown in red. Cells were later fixed, and additional DAPI staining for all nuclei is shown in Fig. 3A and D in blue. AAV infection was performed once on a chip and three times on glass, with consistent results each time. For information about AAV construction and production, please see S2 Text.

### Spontaneous activity demonstrated that culture chambers were connected

**Fig 4:**
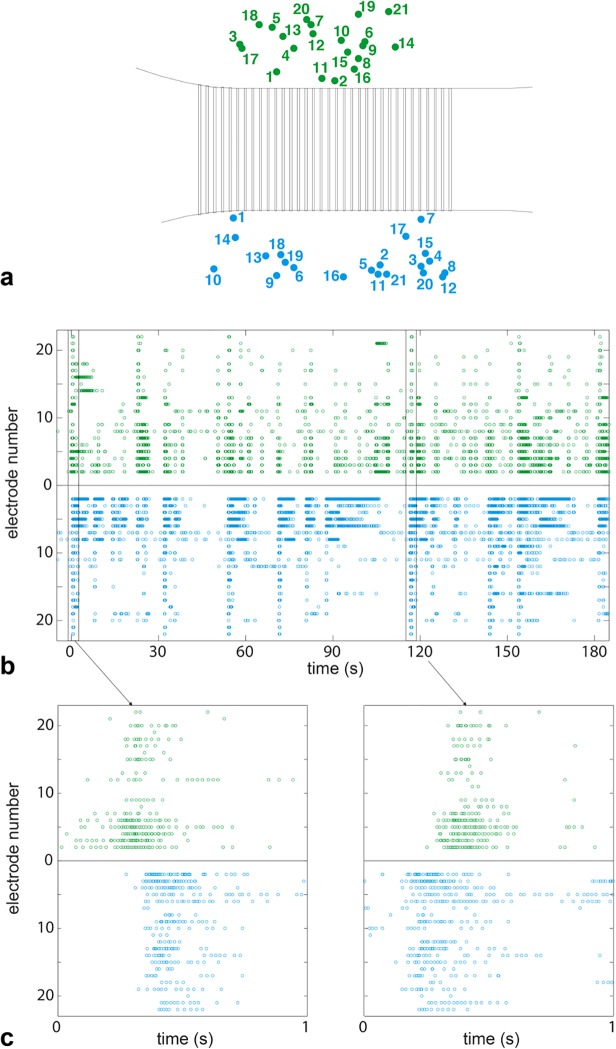
Raster plot showing the two culture chambers were functionally connected. The two culture chambers were connected only by axons in the channels. The top chamber is shown in green, the bottom chamber is in blue. (a) Positions of electrodes on the chip whose signals are displayed in (b) and (c) Inter- and intra-chamber bursts occurred; somas burst together on various time scales with activity propagating from top to bottom (c, left panel) as well as bottom to top (c, right panel). Bursts in the two chambers overlapped 6.2% of the time, while the chance of coincidental overlap was 0.02%, proving that the chambers were connected.

The networks in the top and bottom compartments communicated with one another, while also maintaining local activity that only propagated through parts of the culture. Spontaneous activity of the culture was recorded by using many different electrode configurations, and then the overall activity of the culture was assessed. For further details, see Methods section. Forty-two electrodes that were isolated from one another, and that each recorded a different neuron, were chosen based on large, negative-first (somatic), biphasic spike shapes and above-average spiking rates. In our experience, a large, predominantly negative, biphasic spike seen on one or two (neighboring) electrodes and smaller spikes spread out over one to several adjacent electrodes were associated with a soma, as will be seen in later Figs. [42,48] Additionally, we sometimes observed much smaller spike signals on more distant electrodes from the central one(s), which had a variety of different shapes. These signals were associated with axons outside of channels and had to be averaged in order to isolate them from the noise, in accordance with earlier findings. [15] Fig. 4 shows raster plots on different time scales of the activity of these somas. Fig. 4A shows the positions of the cells on the chip: cells in the top culture chamber are shown in green and those in the bottom chamber are in blue. The numbers and colors correlate to the raster plots in Fig. 4B-C. Bursting occurred on many different time scales, and crossed through the channels, as evidenced by the raster plots. The bottom plots show events initiating in either the top or bottom compartment and propagating into the opposite compartment, consistent with axons that grew in both directions through the channels.

### Somas and axons could be matched using spontaneous activity

**Fig 5:**
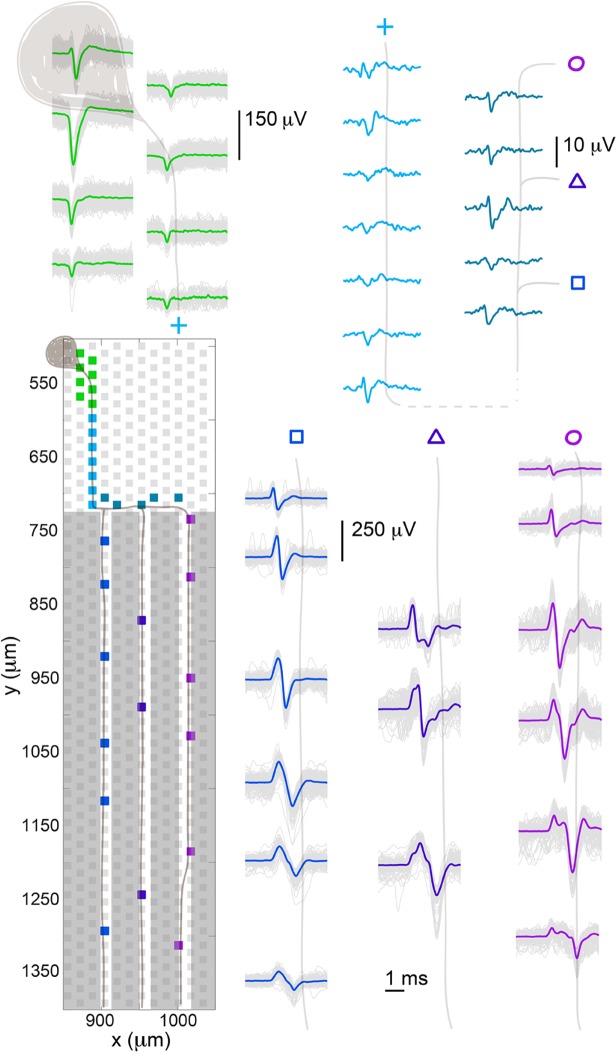
Spontaneous spike propagation from a soma to a branched axon. Spike-triggered averaging of the large green somatic spike revealed a branching axon, which grew into three channels. The footprint of the soma was spread across several electrodes and showed the typical somatic shape: essentially monophasic, negative spike. The small axonal signal outside of the channels is shown in blue. Electrodes in three adjacent channels recorded spikes that were time aligned with that of the soma, and their positions and spike shapes are shown. The spike amplitudes were very different for the different cellular components (soma, axon outside channels, axon inside channels) as indicated by the scale bars. A cartoon neuron was drawn over the traces to guide the eye.

The large number and high density of electrodes made it possible to match signals from a soma to those from its axons. During two minutes of spontaneous firing, the soma, whose spike-triggered signals are shown in green at the top left of Fig. 5, spiked over 550 times, and each of these spikes was tightly correlated with signals on electrodes in the channels: 100% of the spikes recorded from the soma were recorded also on electrodes in the channels (blue traces) within a 3 ms delay. The ‘footprint’ of the soma showing a characteristic somatic signal (almost monophasic, negative-first spike) is shown in green, and the electrodes beneath the soma are also shown in green. The spike-averaged footprint of the small branching axon is shown in lighter and darker blue. The average was created by aligning all spikes on a given electrode to the negative peak on the second electrode in the first channel. The spikes in the three channels are shown on the right side of the Fig. with trace colors corresponding to the electrode locations. All 550+ traces were overlaid, and a median trace was plotted on top of these signals. A cartoon neuron and its branched axon growing into different channels were drawn over the electrodes, and this same neuron was drawn over the traces to guide the eye.

### Spike evolution occurred throughout culture development

**Fig 6:**
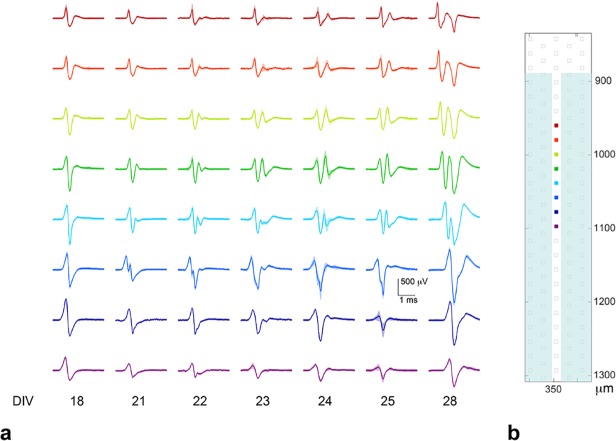
Spike shape evolution on eight electrodes in a channel over the course of 11 days. Signals on electrodes were consistent over short time periods (minutes) but changed dramatically over longer time periods (days). Colors were used to correlate (a) the spike shape with (b) the electrode position in the channel. The red (upper) electrode is closest to the channel entrance. Spike propagation was from the red electrode toward the purple electrode, which was about 200 μm inside the channel. Spike shapes evolved significantly over several days. The simple bi-/tri-phasic axonal signal became a more complicated ‘w’ shape, probably as a result of axonal growth. The mean trace is shown, and the standard deviation of the spikes that were averaged together is outlined in a lighter color.

As the cell culture matured, changes in cell number and distribution, cell excitability, and in spike shape and size occurred. Fig. 6 illustrates the evolution of spike size and shape along eight adjacent electrodes in one channel over 11 days. On the first day (shown in Fig. 6, first column, DIV 18) all the spikes had a similar shape to the one shown in Fig. 5A: a large positive peak followed by a large negative peak, and then a small positive peak. On the fourth day (second column, DIV 21) changes to the spike shape were observed on the electrodes shown in green and blue. By the sixth day (middle column, DIV 23), most of the electrodes recorded spikes with significant positive third phases and developing negative fourth phases. By the eleventh day (last column, DIV 28), the first five electrodes, shown in dark red through light blue, all recorded waveforms that had a tetra- to penta-phasic ‘w’ shape, and spike size on all electrodes had increased. By DIV 37, nine days after the last column shown in Fig. 6, the ‘w’ spike shape was no longer observed, suggesting that the cell responsible for that spike may have died.

### Spontaneous axonal spikes in channels were large in size and unique in shape

**Fig 7:**
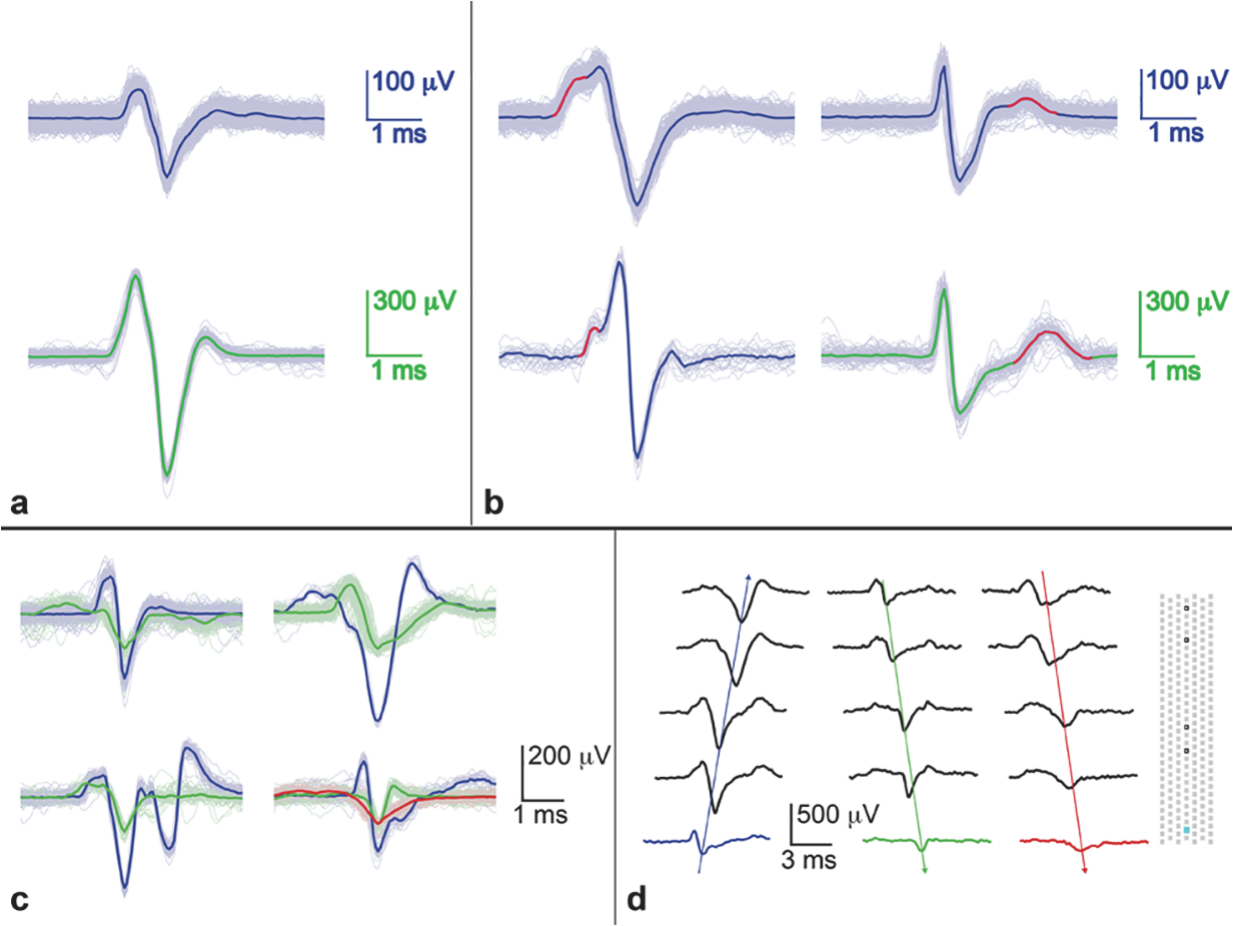
Spontaneous spike shapes on different electrodes in the channels during a single recording session. Spike shapes on a given electrode were remarkably consistent and reproducible over short time periods. (a) The ‘classical’ axonal signal, which is biphasic (blue) or tri-phasic (green), starting with a positive peak, was the shape most often seen. (b) Variations on the classical shape showed either a bump before the first peak (first column) or a bump just after the spike (second column). Colored scale bars correspond to waveforms of the same color. (c) Complex multiple spike shapes in channels. A single spike shape propagated in a single direction while different shapes often propagated in opposite directions. In all cases many trials are shown and median traces are overlaid. (d) Timing of different spike shapes shown in c in the lower right plot; the positions of the electrodes are shown on the right side: the electrode of interest is light blue, and the other electrodes in the channel are outlined in black. Electrode pitch is 17 μm.

The spikes detected in the channels were not only large in amplitude, as Figs. 5 and 6 show, but also had distinct, reproducible shapes, which were consistent on a given electrode for a given time, as Fig. 6 shows. The spikes in Fig. 7A display spike characteristics that are often observed in channels: bi-/tri-phasic axonal signals consisting of a large positive peak followed by a large(r) negative peak, and then (often) a third small positive peak. Fig. 7B shows variations on this theme: the first column shows the same general pattern, but with an additional bump at the beginning of the signal, while the second column shows an additional bump just after the main spike.

More complex spike shapes were also recorded on electrodes in channels, and they, too, were distinct and reproducible over a given time period. Fig. 7C shows spikes recorded on four different electrodes, which each recorded more than one spike shape. In each case, a single spike shape consistently traveled in a single direction. Fig. 7D shows this timing explicitly for the bottom right spikes in 7c, where three different signals traveled through the channel (blue upwards, green and red downwards). All of the spikes shown in Fig. 7 are from electrodes that were in various places inside the channels. In all cases, raw traces from all spikes detected within a 60-second recording period were plotted on top of each other, and the median trace was overlaid. The spike waveforms were highly consistent and reproducible for a given electrode and corresponded to a single spike shape (7a-b) or to two or three distinct shapes (7c).

**Fig 8:**
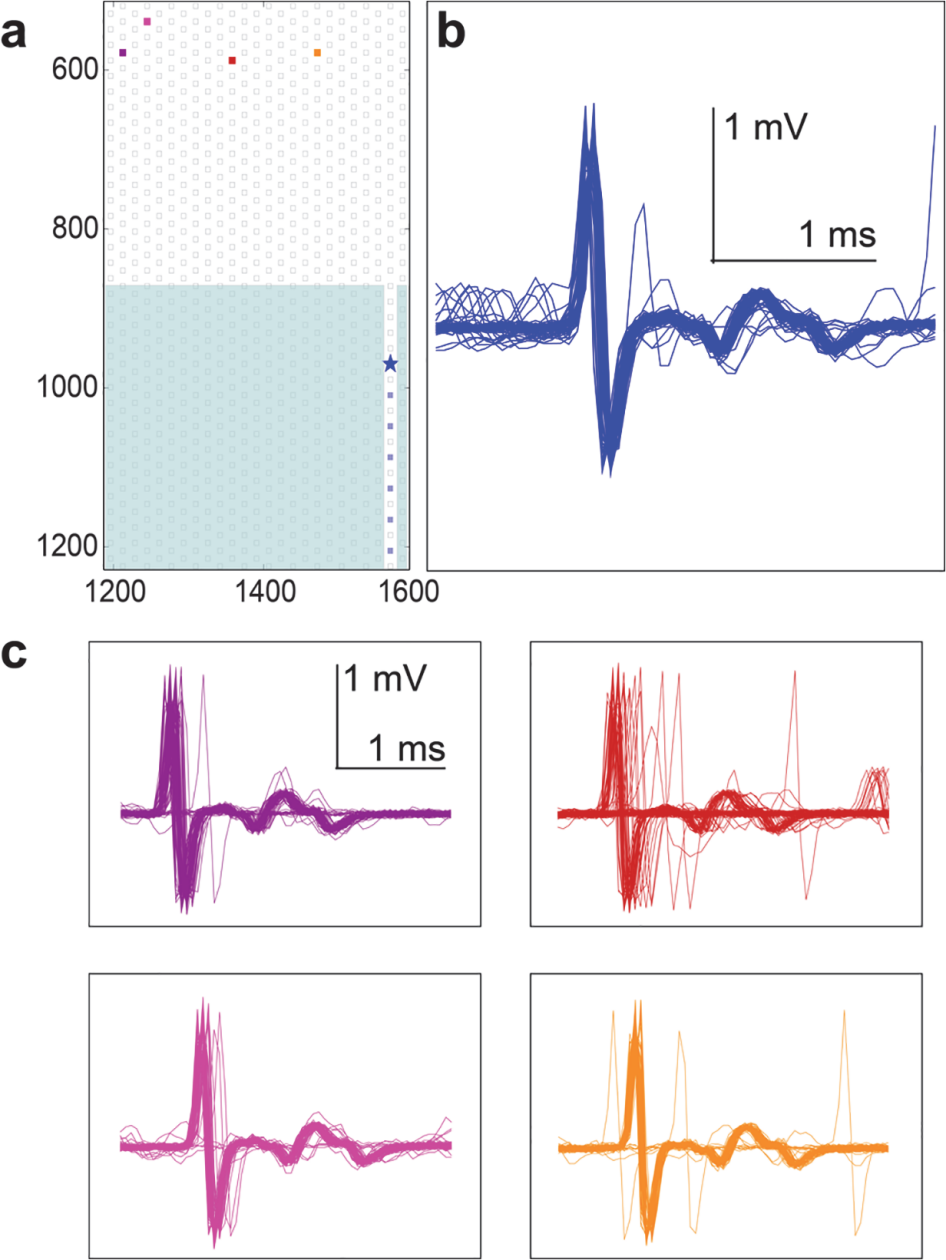
Complicated spike shapes originated from single cells. Complicated spontaneous spikes could be reproduced by stimulations of a single cell at various positions. (a) Positions of electrodes that were stimulated and recorded from. The electrode of interest in the channel, nearest the entrance, is denoted with a star. (b) The spontaneous spike shape on the electrode depicted with a star in a. The large, initial, mostly biphasic spike is followed by a specific and complicated shape. (c) Stimulation at four different sites spanning 250 μm and denoted by the burgundy (left most), pink, red, and orange (right most) electrodes, all elicited the same spike at the electrode of interest in the channel. Latencies varied slightly, and jitter was introduced, but the evoked spike shape remained the same as the spontaneous spike shape.

To find the origins of more complex spikes, electrodes that recorded complicated spike shapes during spontaneous activity were stimulated in order to locate corresponding somas and axons outside of the channels. The matched cell bodies were subsequently stimulated in order to observe the spike shapes in the channels at the electrode in question. One such set of spontaneous and stimulus-evoked spikes is shown in Fig. 8. Four different electrodes spanning 250 μm were all able to elicit the same complicated waveform on the electrode in the channel that had been observed during spontaneous firing (Fig. 8A, marked with a blue star). Stimulations at each one of these four points also reliably evoked spikes at the other three points, consistent with the idea that these electrodes were all stimulating and recording from processes extending from a single cell.

**Fig 9:**
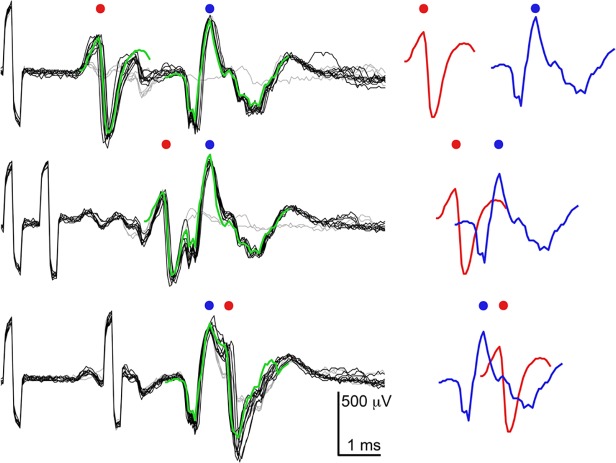
Addition of single spike waveforms from stimulation of two somas reproduces complicated wave shapes. Top trace shows the two stimulations far apart in time, so that the waveforms do not yet overlap. The middle and bottom traces show two different overlaps. The peaks of the individual waveforms are marked with dots corresponding to their color. Addition of the individual waveforms, shown in red and blue on the right, accurately reproduced the shape recorded in the channel as a result of two stimulations. The s-shaped pulses at the beginning of each trace are stimulation artefacts: the first one (the only one shown in the top trace) corresponds to the stimulus that evoked the blue spike, and the second one (not seen in top trace since it would be to the left of the signal shown) corresponds to the red spike. Some stimulations did not evoke spikes, and they are shown in grey.

In order to observe the types of responses that would be produced in the channels when more than one soma was stimulated, multiple somas, whose axonal branches grew into the same channel, were stimulated with different time offsets. Fig. 9 shows three examples of such stimulations in a single channel at different time offsets. The red and blue waveforms are median waveforms produced by single-electrode stimulations (just one of the somas at a time), while the green waveform is the linear superposition of the red and blue waveforms given the time offsets used for stimulation. As Fig. 9 shows, the predicted waveform reproduces the waveform observed upon stimulation of both somas.

### Stimulation in channels evoked many axons

**Fig 10:**
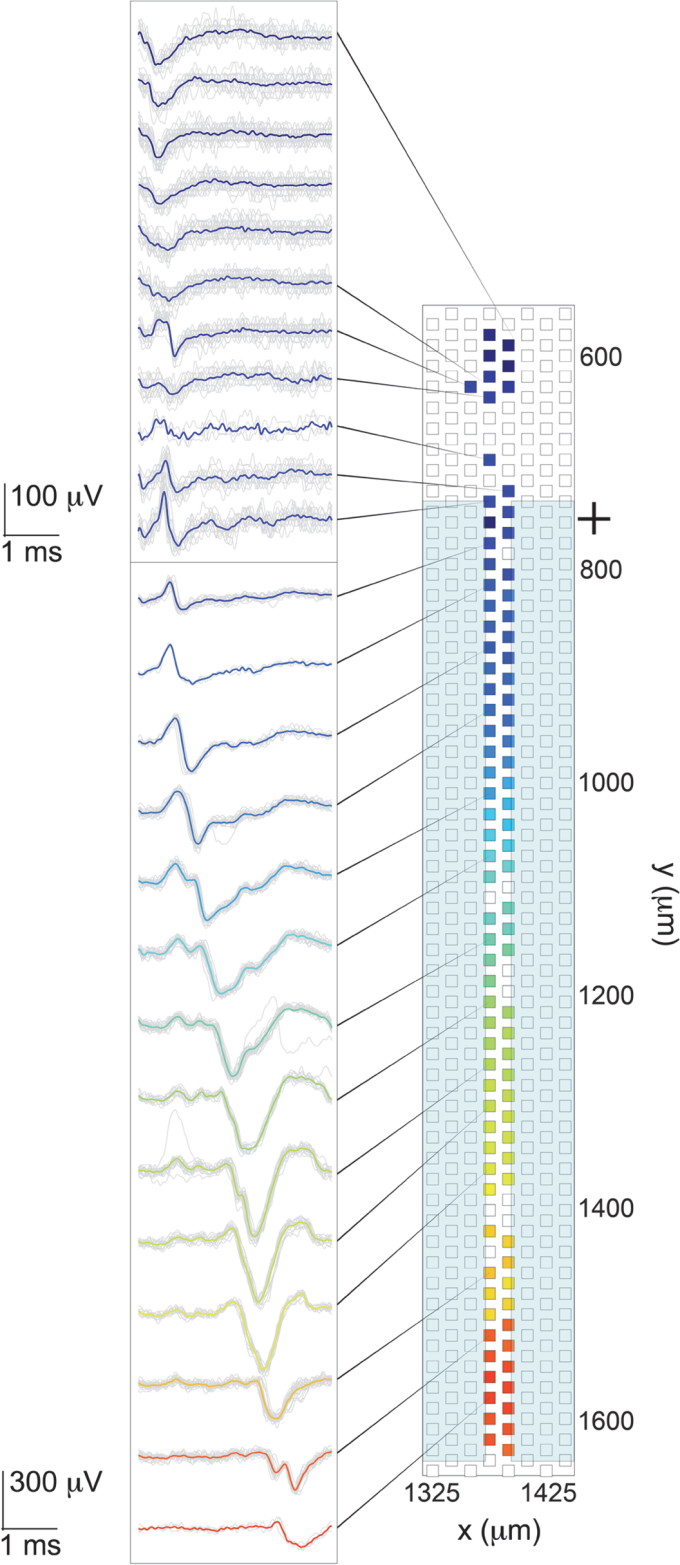
Spike propagation following stimulation in channel. Invasion of an action potential into the soma and subsequent propagation through a channel; colors convey timing information and correspond to the colors of the electrodes depicted on the right. Thirty trials (in grey) for each electrode were overlaid with the median signal. The dark blue somatic spike gives way to a smaller axonal spike, which at first was hard to detect in the noise and was then amplified by the channel structure as it propagated through the channel. Complex spike shapes were more apparent than in the spontaneous case. The initial stimulation site was an axonal branch in the channel just to the right of the one depicted, and is demarked with a black cross.

Fig. 10 depicts the propagation of an action potential that resulted from stimulation in a channel. The elicited spikes can be seen on the left side of the plot, and the electrodes that recorded spikes on the right side. The action potential was elicited in an axon, which then appears to have propagated antidromically to the soma and then down another axonal branch. The somatic signal (negative-first spike) is shown in the top box (dark blue traces), which then became more bi- or tri-phasic in shape as it propagated along the axon outside of the channel. The strong amplification effect of the channel structures can be recognized by the difference in voltage scales between the top box (outside of channel) and the bottom box (inside channel). As a result of the sizeable stimulation artefact in the channel housing the stimulation electrode, it was difficult to observe the immediate effect on electrodes in the same channel. Thirty raw signals were drawn on top of each other in grey and a mean trace was overlaid in color, representing the spike latency. It was possible to compute velocities from several stimulation data sets. We found an average propagation velocity of 0.51 ± 0.1 m s^-1^ (N = 48, based on nine stimulations on five different chips), consistent with unmyelinated axons as expected in this environment.

The S1 Movie shows how extensive the result of stimulation can be. Axonal branches belonging to tens of cells were stimulated inside a single channel and caused the AP to propagate back to cell bodies as well as to other axonal branches. The resulting display is a dynamic portrait of an interconnected region of the culture.

## Discussion

Several different designs for the culture chambers and channels were fabricated in order to test: (i) how well PDMS would seal to the CMOS surface, which has a surface roughness on the order of micrometers, (ii) whether precise alignment of devices would be possible, and (iii) how culture viability might be affected by the devices. In general, as long as the PDMS had been freshly prepared and the bonding of PDMS to CMOS chip was performed under clean conditions, no problems with bonding were observed. Treatment with oxygen plasma, as is often done when bonding PDMS to glass, was not necessary, and actually tended to lessen adhesion strength between the chip and the PDMS piece. Based on the activity of cells on the chip, partial lifting off of the PDMS device was observed on three out of more than 50 chips, but this happened because PDMS pieces older than two weeks had been used. Several slightly different geometries with different culture chamber sizes and channel lengths were tested. Precise alignment of channels to electrodes proved to be very difficult and was, therefore, not pursued. Instead, we chose a channel pitch, which was different from that of the electrodes, thus creating some channels that would not be on top of electrodes, but also eliminating the possibility that all channels would be misaligned, see Fig. 1C. Additionally, while the larger culture chambers (5.2 x 1.6 mm^2^) were more convenient during cell plating, axonal signals could not always be correlated to cell bodies, since many somas were not located on the array. Shrinking the culture chambers (to 2.5 x 1.0 mm^2^) mostly eliminated this problem, but since chamber volumes decreased to 1–2 μL, greater care had to be taken during plating to avoid evaporation. Since culture chambers were open at the top, allowing medium and gas exchange, cell viability was not a problem.

Monitoring the spontaneous electrical activity of the culture validated that axons grew through the channels and created functional connections in both directions. The data shown in Fig. 4 are a subset of one dataset that was analyzed to measure network connectivity. Of the bursts that were detected during a 58 min recording, there were 250 bursts in the top compartment (corresponding to a mean rate of 0.07 Hz) and 194 in the bottom compartment (corresponding to a mean rate of 0.05 Hz). 65 of the bursts overlapped, corresponding to 6.2% of the 58 min recording, using an average burst length of 200 ms. The live time (percentage of total recording time during which bursting occurred) was 1.4% for the top and 1.1%, for the bottom compartment. The chance of coincidental overlap was 0.02%, providing evidence that the compartments were connected [49]. Although this burst analysis was only performed on one culture, the connectedness between chambers was observed in all healthy cultures by other methods. For example, stimulation of axons in the channels, such as can be seen in the S1 Movie, often evoked activity in both chambers and throughout the culture. Based on 54 unique stimulations performed in channels, on 17 different chips, 43 showed robust activity in both chambers. Likewise, correlated spontaneous spiking, especially bursting, in the two chambers was regularly observed during data acquisition (data not shown).

Associating a soma with its axon and axonal branches could be performed in various ways. The data shown in Fig. 5 is the result of a spontaneous scan utilizing configurations of high-density blocks outside of channels and low-density, scattered electrodes inside channels. Data from the ‘somatic’ spike was then used as a virtual trigger to find spikes on other electrodes, so-called spike triggering. Plotting the spike-triggered signals recorded on electrodes in three adjacent channels show that the associated axon branched into at least three branches in three different channels (Fig. 5). Time delays in the axonal signals corresponded to what was expected given propagation velocities that have been measured on the chip: 0.51 ± 0.1 m s^-1^ (N = 48). Another method for obtaining similar results also utilized spike-triggered averaging. Data was taken using many random electrode configurations while keeping electrodes beneath suspected somas fixed in all configurations. This data was first sorted and then time-correlated to create spontaneous spiking profiles that were used to associate somatic spikes to axonal spikes. A third method to associate a soma with axonal branches used stimulus-evoked data, achieved by stimulating axons with the array. The method was to stimulate inside of a channel and record the subsequent activity, which inevitably evoked a number of different somas. In all cases and in all cultures, the somatic footprint had several distinguishing characteristics, most of which can be seen in Fig. 5: a large, predominantly negative, biphasic spike was seen on one or two electrodes while smaller spikes spread out over one to several additional electrodes. Furthermore, there was often a much smaller spike signal (which was generally positive-first but could have a variety of shapes) going off in one or two directions away from the electrode with the largest spike, which could be assigned to axonal segments. We have observed this type of somatic footprint in many different preparations, including slices, retinae, and dissociated cells. [15,42,48] The spike-triggered averaging method, the second method mentioned, was the most reliable and robust of the three, but also required the most computing time and power. As long as the somas that were chosen spiked regularly (with a rate of ∼5 Hz or more), it was always possible to find at least part of the associated axon. Generally it was seen that somas closer to the channels, rather than the ones on the edges of the array, were associated with axons in channels.

Based on their locations and rates of spiking over days and weeks, it was inferred that differentiated neurons tended not to proliferate, while microscopy observations showed that astrocytes continued to divide and grow around and on top of neurons (data not shown). Neurons moved and also died, as evidenced by signals that were recorded first on one electrode, and later on an adjacent one, or by signals that disappeared. Stimulation was also affected: an electrode that was successful at stimulating a cell one day may not have been successful the next day. The PDMS channel device helped to anchor axons, but changes in spike shape and size were seen on a regular basis. Axonal extracellular voltage signals were substantially amplified by the channel structures [34,35,50]. Outside of channels, axonal signals could only be distinguished from noise by averaging tens of traces together [15]. Within channels, single traces were large enough to allow detection of single action potentials in both spontaneous and stimulus-evoked firing.

The channels produced a minimal and predictable distortion to the spike shape, but otherwise simply amplified spike size. The model proposed by FitzGerald et al. fits well to our data [34]. They modeled axonal propagation inside and outside of channels, and their predictions for unmyelinated axons are: amplification of the negative peak, associated with the propagating action potential, and positive phases on either side of the negative peak, from a temporary charge shift into the channel, which is like a standing wave. That is, a non-propagating positive phase whose duration coincides with the entry and exit of the AP to and from the channel. They also predicted uniform amplification along most of the channel, which falls off rapidly near each end. We observed positive phases as a result of the propagation itself [51], see the small axonal signals outside of the channels in Fig. 5, but we also saw the standing wave predicted by FitzGerald. For example, in all three channels depicted in Fig. 5, but most prominently in the right-most channel marked with a circle, there was a positive phased bump ∼2 ms after the positive phase associated with the action potential. Assuming a velocity of 0.5 m s^-1^, which we measured, it is expected that the action potential will travel through the 950 μm length channel in ∼2 ms, which is consistent with the location of the positive bump. The same phenomenon is evident in Fig. 10. In 500 μm length channels, the positive phases were masked by the action potential, since the length between positive phases should be ∼1 ms, which is the same length as the duration of the action potential, see Fig. 6. As a result of the fact that dissociated cell preparations were used, the environment around individual cells and their processes influenced the extracellular signals that were recorded. Proximity to electrodes and other cells and cell processes influenced the size and probably the shape of spikes. Propagating spikes often could not be detected on every consecutive electrode in a channel, which could result from other axons or glial cells already occupying the space. For example, if a cell in the bottom compartment grew an axon into a channel, the signal from that axon might only be recorded on a subset of electrodes in the channel near the bottom compartment, and then the signal might disappear or shrink in size on electrodes closer to the top compartment. This phenomenon could result from the axon having to grow around axons already extended from cells in the top compartment.

The temporal evolution of spike size and shape was observed on a regular basis and took place in many channels in all cultures, although not all electrodes in channels showed developments over short time periods as substantial as those shown in Fig. 6. Generally, spike height, especially the negative peak(s), was observed to increase over time, see also the S1 Fig. Spontaneous spiking data, that were acquired regularly during the life of a culture, showed that channels’ average activity rates and spike heights both increased over time in all cultures that were studied (N = 35) over the one to two months that data were recorded. Further filling of channels by additional neural processes and glia were expected to create signals whose magnitude increased over time [34],[52]. The channels’ increased resistivity via decreased extracellular volume, thus enabled much higher voltage readings. [50],[34] Naturally, spike shapes in channels, as well as characteristic somatic footprints outside of channels, sometimes weakened and disappeared over time, which we attribute to apoptosis.

As we and others have observed, an electrical potential traveling in a volume conductor has a distinct shape when measured extracellularly, which is tri-phasic and starts with a positive-going signal [15]. Traveling signals are asymmetric (the third positive phase is less pronounced) because of the inactivation of Na^+^ channels following an action potential (the refractory period) [51],[53]. These types of axonal signals were the ones most often recorded in the channels, but other shapes were also observed. Bi- and tri-phasic spikes originated from a single cell, and we have observed that the more complicated spikes also originated from a single cell, most likely as a result of axonal branching or turning within a single channel. The waveforms shown in Fig. 8 all have the same shape, which looks like a positive-first axonal spike followed by a characteristic tri-phasic tail. This shape was first observed in spontaneous spikes and later reproduced by stimulating at four different locations. It is very unlikely that two or more different cells, when stimulated, would be able to reproducibly create this type of identical response on the same electrode. Thus we concluded that all four stimulus-points evoked the same cell (cell body and axon) and that such a distinct spike shape represents a signature of a cell at a given time. Aside from the data shown in Fig. 8, several other unusual spike shapes on different electrodes were examined in a similar fashion, including the ‘w’ shape shown in Fig. 6. In all cases, the complicated spike shapes could be induced by stimulation of a single electrode, which could be attributed to one soma, or by multiple electrodes, attributed to a single cell, just like the data shown in Fig. 8. Based on these findings, and the observation that single action potentials add linearly on electrodes in the channels (see below), it is believed that the strange shapes were recorded from single axons that had turned or branched inside of a channel. Activity in different parts of the same axon will be time-locked and correlated to the electrical activity of the respective soma, so that the individual spikes, picked up on the same electrode, will have a complex shape. Although more difficult to observe, complex waveforms, including ‘w’ shapes and other shapes with large positive phases, were observed outside of channels as well, and, in cases where visualization of lipofected neurons was possible, these shapes were observed in the vicinity of axonal branches.

The lower limit of the channel width, 2 μm, results from the fabrication technique and tools available. Slightly thinner channels (0.5–1 μm) could be fabricated using more elaborate cleanroom techniques, or even nanochannels could be created using suitable techniques. [54] The resulting small-diameter channels could, however, be prone to clogging during functionalization, and later inhibit medium exchange, especially following axonal outgrowth. A large multiplicity of very-small-diameter channels would poorly match the HDMEA, since several channels would cross over a series of the same electrodes. A smaller number of channels, e.g., matched in number to the electrodes, might inhibit axonal outgrowth into channels as a result of the large separation between channel openings. The result of the 2–12 μm channel width is that many axonal branches from several or many different cells grow into a single channel. Due to our ability to assign even complex spike shapes to single cells, as a consequence of the many available electrodes, it is possible to distinguish spikes of different cells. In fact, it is possible to sort spikes based on shape within channels and then to associate those spikes with single somas outside of channels based on timing information (data not shown). Since spikes in channels add linearly to one another, it is even possible to do this when spikes overlap in time. These features mean that spike sorting and signal analysis is possible even on a larger cohort of axons in a small space.

There have been speculations about the possibility of observing ephaptic coupling between axons (electric field proximity effects not associated with chemical or electrical synapses) in a channel as a result of the close confinement of axons and the large extracellular spikes. [35,39] We performed several experiments, also in very narrow channels (2–5 μm), in an attempt to elicit ephaptic effects but have not found any clear evidence for ephaptic coupling. Several somas whose axons grew into a single channel were stimulated with varying time offsets. Although stimulations were successful, the resulting spikes in channels were simply the superposition of individual spikes overlapped in time, see Fig. 9. Such experiments were performed at multiple sites on three different chips, and the results were consistently like those shown in Fig. 9: aside from minor differences due to jitter, direct superposition of single waveforms could always be used to reproduce the waveforms produced by doing double stimulations. Ephaptic coupling may be a very local effect that does not occur between all axons in a single channel, but perhaps only between adjacent or overlapping axons, thus making the effect quite subtle. Since culture chambers were larger than the active array, not all somas whose axonal signals were recorded in channels could be recorded from or stimulated. Also, some somas simply did not respond to stimulation. It may be possible that ephaptic effects could be observed in even more confined environments, with more judiciously chosen somas, or by employing more exhaustive stimulation protocols.

Stimulation site and strength were very important in determining the amount of activity elicited by a pulse. A single stimulation could evoke varying amounts of activity depending on the number of axons stimulated. As stimulation voltage was increased, a larger number of axons was recruited [44,55], which resulted in more extensive activity. A movie demonstrating one such stimulation event is included in the supporting information (S1 Movie). The stimulating electrode is demarked with a cross; see Methods section for details of how movies were made. Since only a subset of electrodes could be read out at once, in order to create such a movie, about 3000 stimulations at the same spot had to be done to evoke and record activity. The resulting movies (over 100 movies have been made from stimulations on more than 20 different chips) are a good way to visualize the data, including the interconnectedness of cells on a chip. Activity was directly evoked and not the result of synaptic transmission as evidenced by the short latency and reproducibility of evoked activity. Stimulation in channels sometimes evoked activity in both culture chambers, and sometimes only in one. Stimulation in culture chambers was observed to evoke activity in the same chamber and in channels and only rarely in the opposite chamber. The small (5.8 x 8.2 μm^2^) electrodes allowed us to stimulate either single cells or a small number of cells in culture chambers. As a result, the evoked activity was spiking of individual cells, rather than a network burst that may result from the simultaneous stimulation of many cells with larger electrodes. Comparison of spontaneous and evoked spikes on the same electrode in a channel showed that evoked spikes often appeared to be mixtures of the spontaneous spikes. This result is in line with the data shown in Fig. 9, where superposition of spikes was observed, following stimulation, to result from several cells firing at the same time.

## Conclusion

A novel platform for studying axonal properties, which combines a high-density MEA with a microfluidic channel device for neuronal cell culture, has been described. Its utility in guiding axons between two chambers of cultured neurons, as well as the strong enhancement given to electrophysiological signals make it a useful tool for studying axonal properties. The high density of electrodes on the HDMEA combined with the signal amplification (by a factor of 20 to 150) provided by the PDMS channels make it possible to read out single action potentials propagating along long lengths of isolated axons at high spatial resolution and to study intricate details of axonal signal propagation.

PDMS channel devices created two culture compartments that were connected only by axons growing through the channels. Within the channels, tens of electrodes recorded propagating spikes and revealed characteristic shapes with minimal deformation. Simple tri-phasic waveforms and more complex shapes were demonstrated to originate from a single cell. Even more complicated wave shapes could be created by stimulating several somas, which resulted in a linear superposition of their spike shapes in the channels. Spike-triggered averaging showed that an axon in a channel could be associated with a soma and the respective axonal segment outside of the channel. Stimulus-evoked firing was advantageous in assigning axons to their respective somas, and was used to recreate waveforms that had been observed during spontaneous firing. Stimulation in channels evoked many axons, which then back-propagated to somas in the culture chambers.

Given the recent renewed interest in the axon, both for its information processing capabilities as well as its medical applications, our platform is applicable to a variety of studies. Using the same setup and cells, axonal branches could be studied for differences in propagation velocities, as well as branch point failures and reflections of action potentials at branch points. Additionally, the effects of different types of coupling between axons could be studied, including coupling via gap junctions, axo-axonic (and glial-axonic) synapses, and, possibly, ephaptic interactions. There is increasing evidence that axonal action potential shapes can be modulated [56,57] and that the modulation carries real implications for neurotransmitter release [58]. The ability to follow and perhaps evoke waveforms of different shapes could be very useful in better understanding some of these effects. More elaborate preparations using the same basic tools could be established, such as co-cultures of complementary cells or utilizing fluidic isolation to apply chemicals, viruses, or a specific medium to a single chamber. Although not yet fully exploited, the system has great potential for many different applications.

## Supporting Information

S1 Fig Evolution of spike size and shape in channels over time. Spikes shown in (a) and (b) were from the same electrode and spikes shown in (c) and (d) were from the same electrode, but there was a six day time lapse in between. From a to b, the change was not too drastic, however the size of the spike clearly increased by a factor of two. From c to d, the effect was more dramatic, especially in the blue spike. The positive peak became more complicated (two clear bumps instead of one) and the negative peak increased by a factor of five. Spikes marked in green and blue propagated in the same direction.
(TIF)

S1 Movie Stimulation of axons in a channel back-propagating to somas in chambers and along axonal branches. Colors correspond to spike amplitudes with red representing the most positive (50 μV and above) and blue the most negative (-20 μV and below) voltage amplitudes. The characteristic signature of a propagating triphasic signal was a positive (red) peak followed quickly by a negative (blue) peak, and then another smaller positive peak at the end. The signals were especially large in amplitude in the channels. Outside of the channels, somatic activity was also readily apparent but smaller in amplitude, while axonal signals were yellow in color, and generally hard to detect in the noise.
(AVI)

S1 Text Immunohistochemistry. (DOCX)

S2 Text AAV Construction and Production. (DOCX)
