# Supplementary figures and images for: Recording Large Extracellular Spikes in Microchannels along Many Axonal Sites from Individual Neurons
Source: PLoS One. 2015 Mar 3;10(3):e0118514. doi: 10.1371/journal.pone.0118514 (PMC4348166; doi:10.1371/journal.pone.0118514)

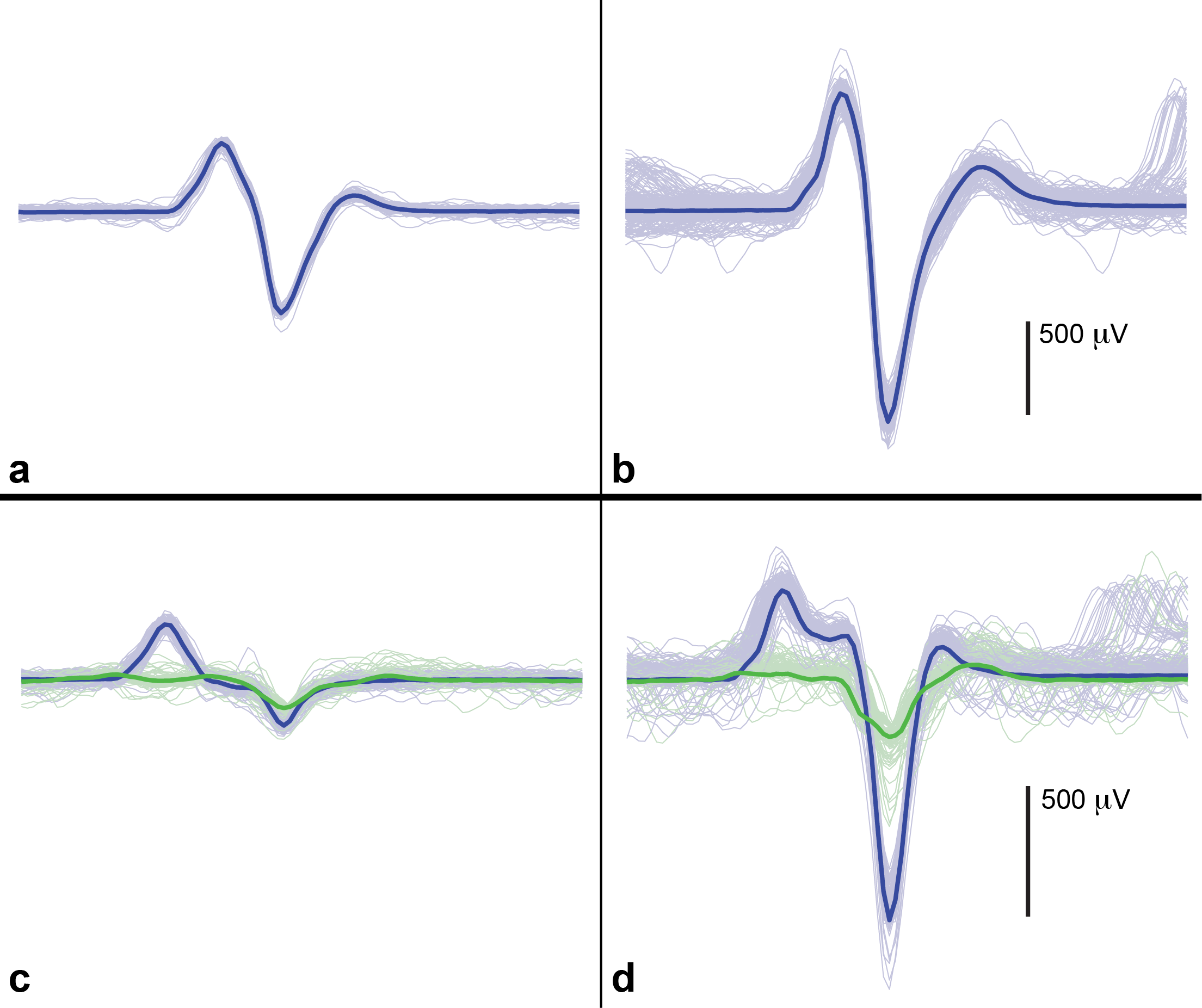

Supplement: S1 Fig — Spikes shown in (a) and (b) were from the same electrode and spikes shown in (c) and (d) were from the same electrode, but there was a six day time lapse in between. From a to b, the change was not too drastic, however the size of the spike clearly increased by a factor of two. From c to d, the effect was more dramatic, especially in the blue spike. The positive peak became more complicated (two clear bumps instead of one) and the negative peak increased by a factor of five. Spikes marked in green and blue propagated in the same direction. (TIF) [file pone.0118514.s001.tif]
