# Supplementary material for: Recording Large Extracellular Spikes in Microchannels along Many Axonal Sites from Individual Neurons
Source: PLoS One. 2015 Mar 3;10(3):e0118514. doi: 10.1371/journal.pone.0118514 (PMC4348166; doi:10.1371/journal.pone.0118514)
Supplement: S1 Text — (DOCX) [file pone.0118514.s003.docx]

**S1 Text. Immunohistochemistry.**

All steps were performed on a laboratory shaker set to the lowest speed in order to facilitate introduction of all chemicals into PDMS channels. Chemicals were purchased from Invitrogen unless otherwise specified. The volume of each chemical dilution used was to 1 mL. Cell medium was removed and cells were washed once in phosphate buffered saline (PBS, Sigma-Aldrich) before being fixed in 4% paraformaldehyde in PBS (pH 7.4) at room temperature for 15 min. Cells were then washed twice with ice cold PBS. In order to access intracellular target proteins, cells were permeabelized with 0.25% Triton X-100 (Sigma-Aldrich) in PBS for 10 min, and then washed three times with PBS for five min each. Unspecific antibodies were blocked by incubating the cells in 1% bovine serum albumin (BSA, Sigma-Aldrich) in PBS + 0.1% Tween20 (Sigma-Aldrich) (PBST) for 30 min. Primary antibodies diluted in 1% BSA in PBST were then introduced, and cells were incubated in this solution overnight at 4 °C. Primary antibodies and dilutions used were anti-MAP2 IgG chicken (1:500, abcam), anti-Tau-1 IgG mouse (1:100, Millipore), and anti-GFAP IgG rabbit (1:100).

The next morning the solution was removed, and cells were washed three times for five min each in PBS. The secondary antibodies with fluorophores were then diluted in 1% BSA in PBS, and cells were incubated in the dark in this solution for one to two hours. Secondary antibodies were all diluted 1:200, and they were: Alexa Fluor 555 goat anti-rabbit IgG, Alexa Fluor 488 goat anti-mouse IgG, and Alexa Fluor 647 goat anti-chicken IgG. The solution was removed and cells were washed three times for five min each with PBS. Finally, cells were incubated for one min with 1 mg mL^-1^ DAPI (double stranded DNA stain), and rinsed twice with PBS. A drop of mounting medium (AF1, citifluor) was then placed onto the cells followed by a coverslip. Samples were stored at 4 °C prior to imaging.
