# Supplementary material for: Recording Large Extracellular Spikes in Microchannels along Many Axonal Sites from Individual Neurons
Source: PLoS One. 2015 Mar 3;10(3):e0118514. doi: 10.1371/journal.pone.0118514 (PMC4348166; doi:10.1371/journal.pone.0118514)
Supplement: S2 Text — (DOCX) [file pone.0118514.s004.docx]

**S2 Text. AAV Construction and Production.**

Membrane-bound tdtomato was constructed via PCR, adding a farnesylation domain from Gap43 (Neuromodulin) to the N terminus (MLCCMRRTKQVEKNDEDQKI). Oligos used were ggatccggatccgctagcgccaccATGCTGTGCTGTATGAGAAGAACCAAACAGGTTGAAAAGAATGATGAGGACCAAAAGATCatggtgagcaagggcgaggag and gaattcgaattcggcgcgccTTACTTGTACAGCTCGTCCATGCCGTAC. PCR fragment was cloned into a pAAV vector via restriction sites AscI and NheI to produce pAAV Ef1alhpa DIO mem-tdtomato. Construct was tested for membrane localization in 293T cells and transfection of mouse brain (data not shown). Due to the tandem nature of tdtomato, a farnesylation domain was also added to the middle of the membrane-tdtomato construct, but that did not affect the fluorophore intensity nor the membrane localization. The cre-GFP AAV was ordered from Penn Vector (AV-1-PV2004).

Production of the AAV encoding membrane-bound tdtomato was performed as previously described by triple transfection of HEK 293T cells with PEI (Polysciences, no.23966) with the tdtomato plasmid, an AAV-helper plasmid encoding Rep2 and Cap of serotype 8 and the pHGTI-Adeno1 plasmid encoding helper adenoviral genes.[1] Vectors were purified using a iodixanol gradient (Sigma, Optiprep). Encapsidated DNA was quantified by TaqMan^©^ RT-PCR (forward primer: GGCTGTTGGGCACTGACAA; reverse primer: CCAAGGAAAGGACGATGATTTC; probe: TCCGTGGTGTTGTCG) after denaturation of AAV particles by Proteinase K. Titer was calculated as genome copies (GC) per ml. Titer for the virus was 1.24 x 10^13^.

Viruses were dropped onto cultures and then incubated for several days prior to imaging.

1. Grieger JC, Choi VW, Samulski RJ (2006) Production and characterization of adeno-associated viral vectors. Nature protocols 1: 1412-1428.
